# Roles of the 2-Oxoglutarate-Dependent Dioxygenase Superfamily in the Flavonoid Pathway: A Review of the Functional Diversity of F3H, FNS I, FLS, and LDOX/ANS

**DOI:** 10.3390/molecules26216745

**Published:** 2021-11-08

**Authors:** Yueyue Wang, Yufeng Shi, Kaiyuan Li, Dong Yang, Nana Liu, Lingjie Zhang, Lei Zhao, Xinfu Zhang, Yajun Liu, Liping Gao, Tao Xia, Peiqiang Wang

**Affiliations:** 1 College of Horticulture, Qingdao Agricultural University, Qingdao 266109, China; wyytea2021@163.com (Y.W.); lkytea@163.com (K.L.); 13569718312@163.com (D.Y.); lnntea@163.com (N.L.); zhaolei_tea@163.com (L.Z.); zxftea@163.com (X.Z.); 2 State Key Laboratory of Tea Plant Biology and Utilization, Anhui Agricultural University, Hefei 230036, China; shiyufeng94@163.com; 3 School of Life Science, Anhui Agricultural University, Hefei 230036, China; lingjiezhang16@163.com (L.Z.); liuyajun1228@163.com (Y.L.)

**Keywords:** 2-OGDs, oxidation reactions, functional redundancy, evolutionary relationship, flavonoid pathway

## Abstract

The 2-oxoglutarate-dependent dioxygenase (2-OGD) superfamily is one of the largest protein families in plants. The main oxidation reactions they catalyze in plants are hydroxylation, desaturation, demethylation, epimerization, and halogenation. Four members of the 2-OGD superfamily, i.e., flavonone 3β-hydroxylase (F3H), flavones synthase I (FNS I), flavonol synthase (FLS), and anthocyanidin synthase (ANS)/leucoanthocyanidin dioxygenase (LDOX), are present in the flavonoid pathway, catalyzing hydroxylation and desaturation reactions. In this review, we summarize the recent research progress on these proteins, from the discovery of their enzymatic activity, to their functional verification, to the analysis of the response they mediate in plants towards adversity. Substrate diversity analysis indicated that F3H, FNS Ⅰ, ANS/LDOX, and FLS perform their respective dominant functions in the flavonoid pathway, despite the presence of functional redundancy among them. The phylogenetic tree classified two types of FNS Ⅰ, one mainly performing FNS activity, and the other, a new type of FNS present in angiosperms, mainly involved in C-5 hydroxylation of SA. Additionally, a new class of LDOXs is highlighted, which can catalyze the conversion of (+)-catechin to cyanidin, further influencing the starter and extension unit composition of proanthocyanidins (PAs). The systematical description of the functional diversity and evolutionary relationship among these enzymes can facilitate the understanding of their impacts on plant metabolism. On the other hand, it provides molecular genetic evidence of the chemical evolution of flavonoids from lower to higher plants, promoting plant adaptation to harsh environments.

## 1. Introduction

Flavonoid compounds are a class of important secondary metabolites, widely distributed in the plant kingdom. Different plant species present their characteristic accumulation patterns and specific conjugates [1,2,3]. Flavonoid compounds play important physiological and biochemical roles in various plant organs (leaf, flower, seed, fruit, and root), protecting plants from UV-B irradiation, and have numerous functions during the interactions of plants with the environment, both in biotic and abiotic stress conditions [4,5,6,7].

Flavonoids have a structure consisting of 2-phenyl chromene as the main framework and exhibit the basic skeleton C6–C3–C6. The carbon framework is always modified via acylation, methoxylation, hydroxylation, or *O*-glycosylation of hydroxyl groups as well as C-glycosylation directly on the carbon atoms of the skeleton, catalyzed by serine carboxypeptidase-like (SCPL), *O*-methyltransferase (OMT), 2-oxoglutarate-dependent dioxygenase (2-OGD), and glycosyltransferase (GT) families, respectively [8,9,10]. In addition, the cytochrome P450 hydroxylase and the short-chain dehydrogenase/reductase (SDR) families play a great role during the modification and derivatization of the C6–C3–C6 basic skeleton [11,12]. Therefore, diversified modification has enriched the types of flavonoids (including isoflavones, flavones, flavonols, flavanols, and anthocyanidins) in plants and promoted the adaptation of plants to harsh terrestrial environments.

**Figure 1:**
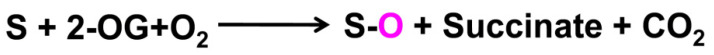
Schematic diagram of the catalytic mechanism of 2-OGDs.The uppercase “S” indicates diverse substrates; S-O indicates the products after oxidation; 2-OG, 2-oxoglutarate.

Cytochrome P450-dependent oxygenases (CYPs) and 2-OGDs are two different classes of oxygenases, catalyzing different oxidation reactions in plant metabolism. CYPs are a kind of heme-thiolate membrane proteins that generally bind to the cytoplasmic surface of the endoplasmic reticulum, while 2-OGDs are non-heme iron-containing soluble proteins that localize in the cytosol. Normally, 2-OGDs catalyze the oxidation of various substrates, requiring 2-oxoglutarate and activated molecular oxygen as co-substrates and ferrous iron Fe (II) as a cofactor. Therefore, in order to improve the reaction efficiency, 2-OGDs activity assays were performed in open tubes while shaking to increase the concentration of active oxygen [13]. In addition, ascorbate was always used as an antioxidant to maintain the ferrous iron state. During the oxidation reactions, oxidized products formed, along with the concomitant decarboxylation of 2-oxoglutarate producing succinate and carbon dioxide (Figure 1).

Members of the 2-OGD family are widely distributed in plants, bacteria, fungi, and mammals [8,14]. The 2-OGD superfamily is one of the largest protein families in plants. The main oxidation reactions catalyzed by 2-OGDs in plants are hydroxylation, desaturation, demethylation, epimerization, and halogenation [8,15,16]. Farrow summarized the functional diversification of 2-OGD in primary metabolic networks (including DNA repair, histone demethylation, and post-translational modifications) and secondary metabolism (including flavonoids, alkaloids, glucosinolates, coumarins, and a variety of plant hormones) in detail [8]. Kawai et al. analyzed the evolution and diversity of the 2-OGD superfamily from green algae to angiosperms (six plant models) [17]. A phylogenetic classification was presented dividing 479 2-OGDs into three classes, designated as DOXA, DOXB, and DOXC. The DOXA class contains the plant homologs of *E. coli*AlkB, which are responsible for DNA repair [14,18]. The DOXB class is involved in proline hydroxylation, a traditional form of post-translational modification (PTM) [19]. The DOXC class is involved in phytohormone metabolism and in the biosynthesis of secondary metabolites, such as flavonoids, alkaloids and terpenoids. It is worth mentioning that the majority of 2-OGDs from land plants are classified into the DOXC class, and their evolution and expansion associated with environmental stresses involve a diversity of specialized metabolites [17].

**Figure 2:**
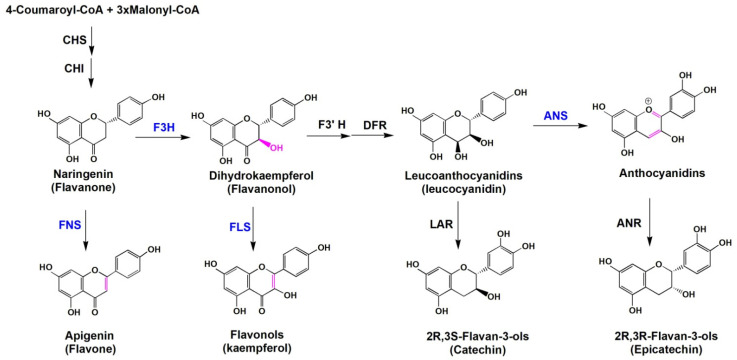
The different 2-OGDs involved in flavonoids metabolic pathway. The blue-labeled enzymes represent the 2-OGDs in flavonoid pathway. The red chemical bonds indicate various types of oxidative reactions. F3H, flavonone 3β-hydroxylase; FNS I, flavones synthase I; FLS, flavonol synthase; ANS, anthocyanidin synthase; LDOX, leucoanthocyanidin dioxygenase; S3H, salicylic acid 3-hydroxylase; DFR, dihydroflavonol 4-reductase; LAR, leucoanthocyanidin reductase; ANR, anthocyanidin reductase; CHS, chalcone synthase; CHI, chalcone isomerase; F3′H, flavonoid 3′-hydroxylase.

Four 2-OGDs catalyze the oxidation reactions involved in hydroxylation and desaturation in the flavonoid pathway, including flavones synthase I (FNS I), flavonone 3β-hydroxylase (F3H), flavonol synthase (FLS), anthocyanidin synthase (ANS)/leucoanthocyanidin dioxygenase (LDOX) (Figure 2). According to phylogenetic classification, FNSI, F3H, FLS, and ANS belong to the DOXC class of 2-OGDs and are responsible for catalyzing the oxidation of the flavonoid “C ring”. A series of oxidation reactions facilitate the formation of different flavonoid subclasses. FNS I catalyzes the oxidation of (2S)-flavonones to yield flavones, which occurs primarily in Apiaceae [20,21,22]. Flavonones can also be hydroxylated by F3Hs at the C-3 position to form dihydroflavonols, participating in the biosynthesis of flavonol and anthocyanin [23,24]. FLSs are responsible for the biosynthesis of flavonol using dihydroflavonols as substrates [25]. ANSs oxidize leucoanthocyanidin to yield anthocyanin monomers (pelargonidin, cyanidin, and delphinidin), which can be further used to produce catechins and anthocyanin glycosides by anthocyanin reductases (ANRs) and UGTs, respectively [26,27].

With the development of molecular biology, more techniques and means are being used to identify the function of genes. Given the importance of 2-OGDs in plant metabolism, their continued functional study in the flavonoid metabolism pathway is indispensable. Some new issues have been mentioned, such as evolutionary relationships, new functions, and functional redundancy among 2-OGDs. In this review, we systematically describe the latest functional research progress regarding the role of the four 2-OGD members F3H, FNS, ANS/LDOX, and FLS in the flavonoid pathway, as well as their evolutionary classification and functional prediction, and discuss recent discoveries in relation to2-OGDs and plant metabolism.

## 2. Catalytic Mechanisms and Adversity Resistance of the Four 2-OGD Members

### 2.1. F3Hs

F3Hs catalyze an early step in the flavonoid pathway, i.e., the formation of (2R, 3R)-dihydroflavonols from (2S)-flavanones (Figure 2), providing precursors for many classes of flavonoid compounds. F3Hs is ubiquitously distributed in plants. From the perspective of evolution of the flavonoid enzymes, F3H is one of the three representative enzymes that were the first to evolve their new function; it followed CHS and preceded CHI [1].

F3H activity was first demonstrated in crude enzyme extracts from flowers of *Matthiolaincana* and subsequently detected in enzyme preparations or cell suspension cultures from other plant species, including parsley and *Petunia hybrida* [28,29,30]. F3H activity could be easily detected in cyanic flower extracts of *Dahlia*, *Streptocarpus*, *Verbena*, and *Zinnia*, but its activity was blocked in special white-flowering mutants of these four species, which indicated F3H activity was indispensable in the anthocyanin biosynthetic pathway [31]. The full-length F3H cDNA was first cloned from *P. hybrida* and expressed in *E. coli*; the recombinant enzyme exhibited high hydroxylation activity, exceeding the activity found in plant extracts [23]. The identification of conserved residues and site-directed mutagenesis analysis confirmed some key amino acid residues as important substrate-binding sites or iron-binding sites in F3Hs [32,33].

Experiments with a maize transposable element *En-[1]*-mutagenized *A. thaliana* population firstly demonstrated that *TRANSPARENT TESTA 6* (*TT6*) encodes *AtF3H*, the only *F3H* gene in the genome [34]. *F3H*, as an early gene of the flavonoid pathway, exhibits a positive relationship with the synthesis of catechins and anthocyanidins [35,36]. F3H plays an important role in plant resistance to biotic and abiotic stresses. Overexpression of *CsF3H* cloned from the tea plant in tobacco increased the content of flavan-3-ols and conferred tolerance to salt stress and fungus infection [37]. Similarly, *A. thaliana* overexpressing *PnF3H* from the bryophyte *Pohlia nutans* showed enhanced salt stress tolerance [38]. Both drought stress and UV-B radiation activated the enzyme RsF3H in the desert plant *Reaumuriasoongorica* and promoted the accumulation of flavonoids, especially anthocyanin [36]. Abscisic acid (ABA) is a vital stress response-associated phytohormone induced by diverse stresses, and *F3Hs* in several species are induced by ABA [39]. The transcript levels of two *F3H* genes in the tea plant (*Camellia sinensis*) and in *Reaumuriatrigyna* were increased under the treatment with ABA [40,41]. Overexpression of *PnF3H* (*Pohlia nutans*) downregulated ABA signaling and increased ROS-scavenging [38].

In order to figure out when and how F3H emerged and evolved, Li Dandan et al. systematically analyzed the function of FNS Is and F3Hs from liverworts, the bryophyte *Physcomitrium patens* (*P. patens*), the lycophyte *Selaginella moellendorffii* (*S. moellendorffii*), gymnosperms, and angiosperms [42]. The most primitive land plants liverworts only produce chalcones, flavanones, and flavones, but no flavonols [43,44]. This is due to the fact that these species possess FNS I but do not express F3H that is required for the production of dihydroflavonols. In *P. patens* and *S. moellendorffii* (which also do not contain *F3H* in their genomes), some dihydroflavonol derivatives could be detected, which resulted from the functional evolution of FNS into a dual-function enzyme with both FNS I and F3H activities. In higher plants—gymnosperm and angiosperm—flavones, flavonols, and anthocyanin are widely distributed, harboring the true F3H genes; specific F3H activity is also achieved, though some gymnosperm F3Hs showed trace FNS I activity. In sum, FNS distributed in primitive land plants is closely evolutionarily connected to F3Hs in seed plants. The functional transitions from FNS I to FNS I/F2H (in liverworts), to FNS I/F3H (in bryophyte and lycophyte), and then to specific F3H (in gymnosperms and angiosperms) provide new sights for the chemical evolution of flavones to flavonols and anthocyanins. The function of FNS I in most angiosperms and gymnosperms is proposed to be replaced by CYP FNS II, though FNS I activities were verified in rice, maize, and *Arabidopsis* [13,45]. F3H performs its dominant function of C-3 hydroxylation for (2S)-flavanones.

### 2.2. FNSsⅠ

Having their substrate in common with F3Hs, FNSs I catalyze the oxidation of flavonones to yield flavones via introducing a double bond between the C2 and the C3 positions. The enzyme activity of FNS was first detected in cell-free extracts of young parsley leaves, and it was proposed that oxygen and Fe2^+^ ions are necessary for the reaction [46,47]. In 2000, the cDNA of FNS I from Apiaceae was cloned for the first time, which enabled further mechanistic studies and the preparative synthesis of flavones in vitro [20]. Gradually, FNSs were cloned and characterized in several other species, including rice, maize, and the liverwort *P. appendiculatum* [13,45,48]. The synthesis of flavones can also be catalyzed by another flavone synthase, FNS II, which is a membrane-bound P450-dependent oxygenase and is ubiquitous in plants [49,50]. This phenomenon is of particular interest from the evolutionary point of view, concerning flavone biosynthesis and functions in plants [51]. FNSs play an important role in the resistance to harsh biotic and abiotic stress. It was reported these two types of FNSs (I and II) in maize can protect plants against UV-B-induced damage by participating in the synthesis of apigenin [50]. Maize ZmFNSI homologs modulated the hypersensitive response (HR) by interacting with nucleotide-binding, leucine-repeat-rich (NLR) proteins [52].

FNSs I in lower/primitive land plants are functionally versatile (Table 1). PaFNS I from liverwort displayed 2-hydroxylation activity, catalyzing the formation of 2-hydroxynaringenin, and flavonol synthase activity, converting dihydrokaempferol (DHK) to kaempferol (K) [48]. FNSs I from *P. patens* and *S. moellendorffii* also possess dual activities (FNS and F3H), converting naringenin into apigenin and DHK [42]. They the key amino acid residue (Tyr240 in PaFNS I/ Pro220 in AtF3H) determining the enzyme activity of FNS and F3H were analyzed and identified [42].

In terms of catalytic mechanism, FNS I presents its own substrate selectivity. It was demonstrated that (2S)-naringenin is a natural substrate for FNS I in vivo, but (2R)-naringenin was not accepted as a substrate [47]. Martens et al. showed that (2R, 3S)-*cis*-DHK is a very poor substrate for PcFLS but is an efficient substrate for FNS I in *Petroselinum crispum* [22]. Conversely, the natural substrates of FLSs, (2R, 3R)-*trans*-DHK and (2R, 3R)-*trans*-DHQ, were not accepted as substrates byFNS Is [47]. F3Hs also exhibit similar substrate selectivity, especially a C-2 selectivity, with only (2S)-naringenin being accepted as a substrate [56]. Therefore, FNS I and F3H may select for substrates with the C-2 α-face B ring. Additionally, FNS I and F3H are known as C-3 β-face oxygenases, indicating that C-3 hydrogens and the C-3 hydroxyl group of their substrates are on the β-face. In contrast, ANS and FLS are C-3 α-face oxygenases.

The model plant *A. thaliana* contains no *FNS*-annotated gene. However, the susceptibility gene downy mildew resistant 6 (*DMR 6*) encodes an enzyme identified as a susceptibility factor to multiple pathogen and bacterial infection in *Arabidopsis* [65]. Inactivation or mutation of the *DMR6* gene in plants can result in resistance to downy mildew [66]. The protein sequence of AtDMR6 has high similarity to that of FNSs I in other species. It was shown that AtDMR6 has FNS I activity, catalyzing the transformation of naringenin to apigenin and that of salicylic acid (SA) into 2,5-DHBA by hydroxylating the C-5 position of SA [13,53]. Therefore, AtDMR6 is known as an FNSI homolog, also called S5H. Recently, two orthologs of *AtDMR6* were characterized in tomato and were named *SlDMR6-1* and *SlDMR6-2* [67]. SlDMR6-1 and SlDMR6-2 display SA-5 hydroxylase activity, converting SA into 2, 5-DHBA in vitro, while in tomato, *SlDMR6-1*, but not *SlDMR6-2*, was upregulated by pathogen infection, and a *Sldmr6-1* mutant displayed enhanced resistance against different classes of pathogens. However, the purified SlDMR6-1 and SlDMR6-2 proteins only displayed S5H activity in vitro and failed to catalyze the conversion of naringenin [67].

Catalyzing the C-5 hydroxylation of SA is a new function of FNS I, which has been verified in *Arabidopsis* and tomato [53,67]. The traditional physiological function of FNS I in most angiosperms is proposed to be lost and replaced by that of CYP FNS II, though the activity of FNS I in rice, maize, and *Arabidopsis* were studied [13,45,68,69,70].

### 2.3. FLSs

FLS is the key enzyme responsible for flavonol biosynthesis in the flavonoid pathway, catalyzing the formation of flavonols from dihydroflavonols.

All land plants, including some lower plants such as bryophytes and ferns, accumulate flavonols, which indicates that these taxa may possess *FLS* genes [71]. However, there are currently no genome reports on these species, and research is mainly focused on other angiosperms or gymnosperms. In 1981, Britsch et al. firstly detected FLS activity in a suspension culture of parsley cells, which could catalyze not only the conversion of flavanone to flavone, dihydroflavonol, and flavonol, but also the formation of flavonol from dihydroflavonol [29]. These reactions require 2-oxoglutarate, Fe2^+^, and ascorbate as cofactors. The first *FLS* cDNA was cloned from *Petunia hybrida* and was validated by heterologous expression in yeast; antisense expression of *PhFLS* in *Petunia hybrida* markedly limited the synthesis of flavonols in petals [54]. Since then, more FLS genes have been cloned and identified in different plants. The first genomic clone encoding FLS was isolated from *Arabidopsis* in 1997; it showed a high level of homology to *PhFLS*, the expression pattern of which was identical to those of three other flavonoid genes (*CHS*, *CHI*, and *F3H*) [72]. In fact, *A. thaliana* genome possesses six *FLS* genes (*AtFLS1–6*), but only AtFLS1 and AtFLS3 have FLS activity, influencing the flavonol levels and the root gravitropic response under abiotic stress; FLS2, 4, and 6 appear to be pseudogenes [25].

In vitro experiments using recombinant FLS proteins from different species (*Citrus unshiu*, *Zeamays*, and *Camellia sinensis*) showed that DHK was a preferred substrate for FLS enzymes, compared with dihydroquercetin (DHQ) [55,73,74]. Homologous or heterologous transgenic works were performed to further verify the function of *FLS* in plants. Overexpression of *ZmFLS1* or *BnFLS* partially restored the flavonol deficiency of *A. thaliana fls1* mutant and recovered the accumulation of anthocyanins to a normal level [74,75]. The antisense expression of *FLS* genes in lisianthus (*Eustoma grandiflorum Grise.*) and *Petunia hybrida* significantly reduced the contents of flavonols and increased the contents of anthocyanins compared to the untransformed plant [54,76]. Overexpression and gene silencing experiments demonstrated that the competitive relationship between McDFR and McFLS was important for anthocyanin and flavonol synthesis in Crabapples [77]. Similarly, heterologous overexpression of *FLSs* in tobacco promoted flavonol biosynthesis and inhibited anthocyanin accumulation; overexpression of *DFRs* in tobacco downregulated the expression of endogenous *NtFLS* and promoted anthocyanin synthesis [78]. These results indicate that FLSs and DFRs regulate the accumulation of flavonols and anthocyanins by competing for their common substrates dihydroflavonols.

In addition to catalyzing the synthesis of flavonols from dihydroflavonols, FLS can accept a variety of other flavonoids as substrates (Table 1). FLS and ANS are highly similar at the polypeptide level, and both ANS and FLS have been found to react with the leucoanthocyanidins [58]. However, FLS only catalyzed the formation of the *trans*-isomer of leucoanthocyanidins, i.e., (2R, 3S, 4R)-*trans*-leucocyanidin. Both the natural substrate (2S)-naringenin and the unnatural substrate (2R)-naringenin could be accepted by FLS [57]. As for the dihydroflavonol substrates, both FLS and ANS showed a preference for the (2*R*, 3*R*)-*trans*-stereochemistry, i.e., (2R, 3R)-*trans*-DHQ. For instance, recombinant OsFLS exhibited both FLS and F3H activities, converting DHQ and DHK into quercetin (Q) and K and naringenin and eriodictyol into DHK and DHQ [79]. *CuFLS* from *Citrus unshiu* showed an additional nonspecific activity consisting in the *trans*-hydroxylation of the natural (2S)-naringenin and the unnatural (2R)-naringenin at the C-3 position [56]. In plants, Owens et al. found that FLS and ANS partially complemented the *Arabidopsistt6* mutants, in which only a small amount of proanthocyanidins (PAs) was deposited in the seed coats [24].

### 2.4. LDOXs/ANSs

ANS is an important enzyme responsible for the synthesis of anthocyanins monomers in the flavonoid metabolic pathway. It catalyzes the production of anthocyanins from leucoanthocyanins via stereo-selective C-3 hydroxylation; therefore, it was named ANS. In terms of the catalytic mechanism, ANS belongs to the class of dioxygenases, so it is aleucoanthocyanidin dioxygenase [80]. Therefore, most researchers think that ANS and LDOX are the same enzyme.

It is thought that the products of ANS are anthocyanidins in vivo. In fact, the initial oxidation product of leucoanthocyanidin is a cyanidin intermediate, flav-2-en-3, 4-diol. Kazuki Saito reported that ANS firstly mediate the oxidation of (2R, 3S, 4S)-leucocyanidin by dehydration to yield 2-flaven-3, 4-diol, which is followed by glucosylation at the C-3 position by 3-UGT [27]. In vitro, 2-flaven-3, 4-diol was trapped in the active site of ANS and further oxidized to Q [27,60]. In *Arabidopsis*, LDOX could complement the *tds 4* (tannin-deficient seed 4) mutant lacking anthocyanin and PA, which proves LDOX was involved in the synthesis of anthocyanins and PAs [59]. However, in vitro studies on ANS using *cis*- and *trans*-leucocyanidin as substrates demonstrated that the stereochemistry at the C-4 position of the leucocyanidins directly affected product selectivity [60]. After incubation of (2R, 3S, 4S)-*cis*-leucocyanidin (a natural substrate) with ANS, Q was the major product (85%), and DHQ (13%) and cyanidin (2%) were minor products; when using (2R, 3S, 4R)-*trans*-leucocyanidin (a chemically synthesized substrate) as a substrate, the product distribution was DHQ (66%), Q (30%), and cyanidin (4%). In summary, the reaction produced mainly DHQ, along with quercetin, but only traces of the anticipated product cyanidin in vitro [60]. Though ANS is called anthocyanin synthase, the function of ANS is not limited to catalyzing the synthesis of anthocyanins. In vitro experiments showed recombinant ANS can perform the activity of FLS, catalyzing the transformation of dihydroflavonol into flavonol and also the formation of cyaniding and PA-dimer from (+)-C [61,63,81]. Both ANS and FLS have also been found to react with naringenin, including natural (2S)-naringenin and unnatural (2R)-naringenin [56,62]. Therefore, compared with FNS I and F3H, ANS and FLS display broader substrate selectivity (Table 1).

Regarding the latest research progress on ANS, researchers identified the function of a new type of LDOX and ANS in the model legume *Medicago truncatula* [64]. This gene, *MtLDOX*, shares low similarity (only 40%) with *MtANS*. Recombinant MtLDOX and MtANS can both catalyze the production of anthocyanins from leucoanthocyanidins. In addition, they can also use (+)-C as a substrate to produce cyanidin. Enzyme kinetic experiments showed that MtLDOX has higher affinity than MtANS for the substrate. However, their physiological function in *Medicago truncatula* is different. The alfalfa seedlings of an *ans* mutant lost the function of synthesizing anthocyanins, while the mutation of MtLDOX had almost no effect on anthocyanin accumulation in the seedlings. As we all know, PAs are primarily composed of different starter units and extension units. By detecting the types of PAs in the seeds of *ldox* and *ans* mutants, most PAs in the *ldox* mutants appeared polymerized with (+)-C as the starter unit, and the PAs in the *ans* mutants were mostly polymerized with C-type carbocation as the extension unit, while in the PAs in the *ans/ldox* double mutants, both the extension unit and the starter unit were exclusively C-type catechins.

By analyzing the above mentioned experimental results, we drew the following conclusions: firstly, in *Medicago truncatula*, ANS mainly controls the biosynthesis of anthocyanins and also determines that the PA extension units are EC-type catechins during the polymerization process; secondly, LDOX can catalyze the conversion of (+)-C to cyanidin, but the mutation of *LDOX* in plants does not affect the accumulation of anthocyanins, indicating this metabolic flux is not the main pathway for the synthesis of anthocyanins. The authors believe that LDOX determined the presence or absence of (+)-C in alfalfa, since, when *LDOX* was mutated, a large amount of (+)-C accumulated, and the content of EC was greatly reduced. They also indicate that in other plants, such as *Lotus corniculatus*, *Gossypium hirsutum*, and *Desmodium uncinatum*, the dominant catechin monomer is (+)-C. The authors speculate that the reason for this phenomenon is that no homologous gene of this new “LDOX” is present in the genome of these plants.

**Figure 3:**
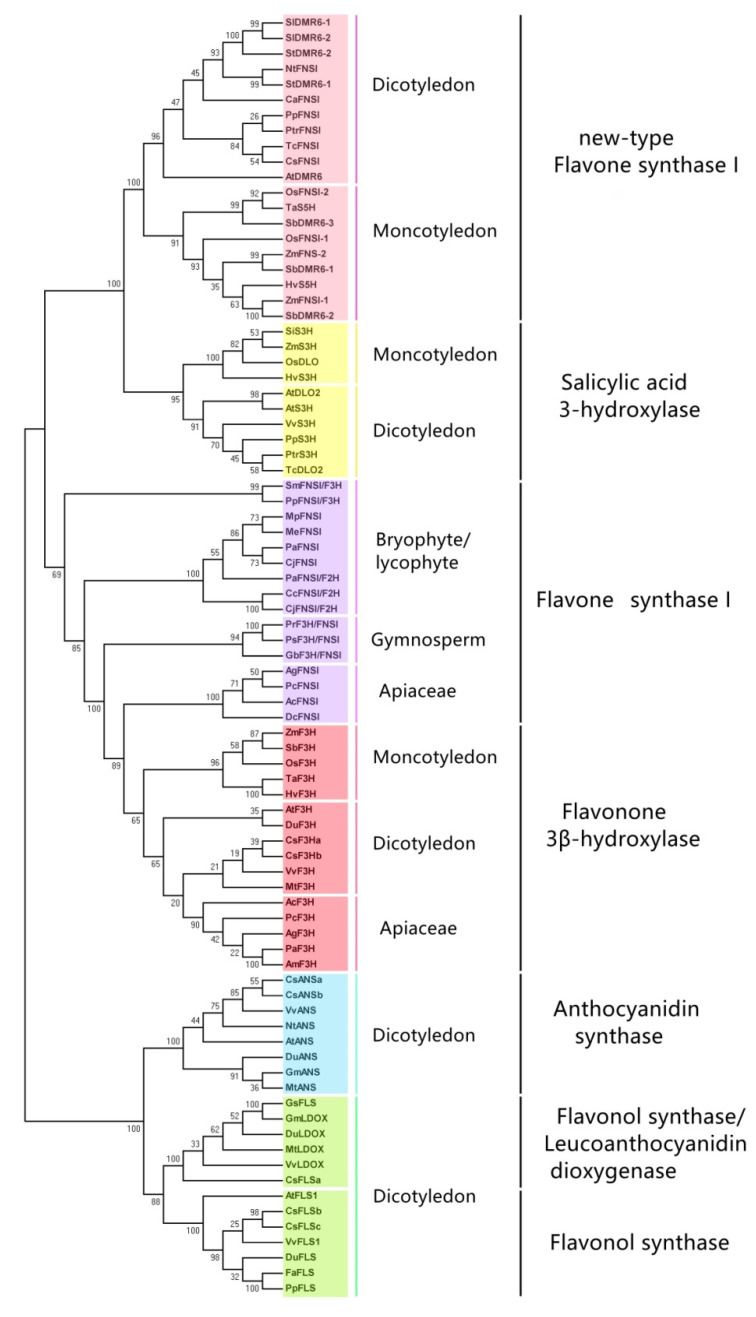
Phylogeny of F3H, FNS Ⅰ, ANS/LDOX, FLS, and S3H proteins from different species.

Careful analysis and comparison of the sequences of MtLDOX and MtANSrevealed that they are two completely different types of 2-OGDs, though they were both named LDOX. The phylogenetic tree showed that MtLDOX is closer to the FLS clade (Figure 3). We speculate it is a new-type of LDOX, which can catalyze leucoanthocyanidins, dihydroflavonols, and (+)-C (Table 1).

These functionally known 2-OGD homolog proteins were collected from different plant species, including monocotyledons, dicotyledons, gymnosperms, bryophytes, and liverworts. Aphylogenetic analysis was performed using the neighbor-joining method with 1000 bootstrap replicates, by MEGA version 5.0. The numbers indicate the confidence percentages. The accession numbers of the protein sequences obtained from GenBank and the plant genomics resource Phytozome 13, are summarized in Supplementary Table S1.

## 3. Phylogenetic Analysis of Plant 2-OGDs

In order to further understand the evolutionary relationship and functional differentiation of the four 2-OGDs in the flavonoid pathway, a phylogenetic tree was established with different 2-OGDs from diverse species including monocotyledons and dicotyledons (Figure 2). To understand the complicated evolutionary processes of FNS I (or S5H), the FNSs I from gymnosperms, bryophytes, and liverworts were added in this phylogenetic tree. To overcome the functional redundancy of these enzymes and distinguish their functions, another 2-OGD salicylic acid 3-hydroxylase (S3H) was also introduced in this tree. These five classes of enzymes were divided into two distinct clades, one consisting of FLSs and ANSs, and the other consisting of F3Hs, FNSs I, and S3Hs. Phylogenetically, FNS I is closely related to S3H and F3H, and FLS and ANS are closely related.

It is worth noting that the FNSs I divide into two branches. Among them, the typical Apiaceae FNSs I are clustered with F3Hs, away from other angiosperms’ FNSs I (also named S5H and DMR6). The FNSs I in Apiaceae plants show a high level of sequence identity with F3Hs. Cheng mentioned the Apiaceae FNS I evolved from an ancestral F3H via a duplication event and arose much later than F3H [82,83]. The function involved in flavone synthesis of FNSs I in dicotyledons and monocotyledons is rarely studied and appears to be confined to the Apiaceae family. Only a few FNSs I from non-Apiaceae species including rice, maize, and *A. thaliana* were studied in detail [13,45]. However, in this branch, both AtDMR6 and SlDMR6 displayed SA-5 hydroxylase activity, with no FNS activity [67]. In addition, the FNS I branch is more closely related to S3Hs, which also form a distinct subgroup. Though there is functional redundancy between S3H and S5H, for example, S3H can catalyze SA to produce 2,3-DHBA and 2,5-DHBAin vitro and can also complement the *dmr6* mutants [84,85], S3H and FNS do not overlap in the phylogenetic tree, indicating that these two classes of 2-OGDs have their own characteristics in the evolution of amino acid residues. Therefore, it is speculated that FNSs I in higher plants mainly perform the hydroxylation of SA, and the activity of flavone synthesis may be substituted by FNSs Ⅱ. We named the FNSs I in higher plants (from non-Apiaceae species) as new-type FNSs I (Figure 3). The phylogenetic tree also shows that the FNSs I from lower plants (liverworts, bryophytes, lycophytes, and gymnosperms) seem to have originated prior to F3Hs, differently from Apiaceae FNSs I. Li Dandan analyzed the evolutionary relationship between FNS I and F3Hs and hypothesized that in liverworts, FNS I might serve as the ancestor of seed plant F3H [42].Through systematically analyzing the function and evolutionary relationship of FNS I and F3Hs from different phyla, it has been proposed that ancestral forms of 2-OGD enzymes are often functionally promiscuous and display broader substrate specificity than more evolved forms [86].

Analyzing the FLS and ANS clade, it is worth noting that some of the enzymes annotated as LDOX are clustered with FLS. A careful analysis of the FLS group indicated it is divided into two branches, one of which includes all FLS enzymes, and the other contains both FLS and LDOX, including GsFLS (*Glycine soja*), CsFLSa (*Camellia sinensis*), VvLDOX (*Vitis vinifera*), DuLDOX (*Desmodium uncinatum*), MtLDOX (*Medicago truncatula*), GmLDOX (*Glycine max*). The MtLDOX we mentioned earlier belongs to this blended branch [64]. MtLDOX could convert leucocyanidin to cyanidin and (+)-C to cyanidin, and (+)-DHQ to Q, only the latter activity being shared with FLS, indicating functional redundancy or incomplete evolution of substrate selectivity among these proteins. This implies that the enzymes in this branch have similar activity, which enriches the functions of FLSs.

## 4. Conclusions and Prospects

With the popularity of high-throughput sequencing technologies, including transcriptomics and genomics, an increasing number of 2-OGD genes have been identified in various species. The functions of the four members of the 2-OGD family examined in this article in the flavonoid pathway have also become clearer. Substrate diversity analysis indicated that F3H, FNS Ⅰ, ANS, and FLS perform their respective dominant functions in the flavonoid pathway, despite functional redundancy among them. The phylogenetic tree showed that two types of FNS Ⅰ were classified. The FNSsⅠ in Apiaceae species, bryophytes/lycophytes, and gymnosperms mainly perform FNS activity, while the new-type FNSs Ⅰ in angiosperms may mainly display the activity of C-5 hydroxylation of SA. Therefore, it is valuable to discuss the balance of roles played by FNS Ⅰ and FNS Ⅱ in plants. Additionally, a new class of LDOXs was discussed, which is closer to the FLS clade and exhibited LDOX/FLS activity. These LDOXs can catalyze the conversion of (+)-C to cyanidin, further influencing the starter and extension unit composition in PAs. Therefore, whether possessing a homologous gene of the new-type *LDOX* can help judge the differences in the types (*cis/trans*) of catechin accumulation in plants. The altered enzymatic function may provide the molecular genetic evidence for the chemical evolution of flavonoids from lower to higher plants, promoting plant adaptation to harsh environments.

## Figures and Tables

**Table 1:** Summary of the substrates and products catalyzed by the four examined members of the 2-OGD superfamily, involved in the flavonoid pathway and verified in vivo and in vitro.

The Members of 2-OGDs	Substrates	Corresponding Product	Verified in Vivo or in Vitro	References
F3H	(2S)-Flavanone	(2R, 3R)-Dihydroflavonols	in vivo and in vitro	[23,31]
FNS	(2S)-Flavanone	Flavones	in vivo and in vitro	[20,47]
	(2R, 3S)-*cis*-DHK	Kaempferol	in vitro	[22]
	Salicylic Acid	2,5-DHBA	in vivo and in vitro	[53]
FLS	(2R,3R)-*trans*-Dihydroflavonols	Flavonols	in vivo and in vitro	[54,55]
	(2S)-Naringenin	(2R,3S)-*cis*-DHK, (2R,3R)-*trans*-DHK, Kaempferol and Apigenin	in vitro	[56,57]
	(2R)-Naringenin	(2S,3S)-*trans*-DHK	in vitro	[56]
	(2R,3S,4R)-leucocyanidin	DHQ, Q and Cyanidin	in vitro	[58]
ANS/LDOX	(2R,3S,4S)-leucocyanidin	(4S)-flav-2-en-3,4-diol, Cyanidin	in vivo	[27,59]
	(2R,3S,4S)-leucocyanidin	Q (85%), DHQ, and Cyanidin	in vitro	[60,61]
	(2R,3S,4R)-leucocyanidin	DHQ (66%), Q (30%) and Cyanidin	in vitro	[60]
	(2R,3R)-*trans*-Dihydroflavonols	Flavonols	in vitro	[61]
	(2S)-Naringenin	(2R,3S)-*cis*-DHK, (2R,3R)-*trans*-DHK, Kaempferol and Apigenin	in vitro	[58]
	(2R)-Naringenin	(2S,3S)-*trans*-DHK	in vitro	[62]
	(+)-Catechin	Cyanidin	in vitro	[63]
New-type LDOX	(2R,3S,4S)-leucocyanidin	Cyanidin and Q	in vitro	[64]
	(+)-Catechin	Cyanidin	in vivo and in vitro	[64]
	(2R,3R)-*trans*-Dihydroflavonols	Flavonols	in vitro	[64]

## Data Availability

Not applicable.

## Supplementary Materials

The following are available online. Table S1. The sequence information used in the phylogenetic tree.

## Abbreviation

PAs: proanthocyanidins; DHQ, dihydroquercetin; DHK, dihydrokaempferol; Q, quercetin; EC, Epicatechin; C, Catechin; SA, salicylic acid; 2,5-DHBA, 2,5-dihydroxybenzoic acid; 2,3-DHBA, 2,3-dihydroxybenzoic acid.
