# Supplementary material for: Roles of the 2-Oxoglutarate-Dependent Dioxygenase Superfamily in the Flavonoid Pathway: A Review of the Functional Diversity of F3H, FNS I, FLS, and LDOX/ANS
Source: Molecules. 2021 Nov 8;26(21):6745. doi: 10.3390/molecules26216745 (PMC8588099; doi:10.3390/molecules26216745)
Supplement: Supplementary file 1 [file molecules-26-06745-s001.zip › molecules-1447977-supplementary.pdf]

Supplementary Table S1. The sequence information used in the phylogenetic tree.

Group	Designation	Match in NCBI	Description
New-type FNSI	**SlDMR6-1**	XP_019069725.1	*Solanum lycopersicum*
	**SlDMR6-2**	XP_004241427.1	*Solanum lycopersicum*
	**StDMR6-1**	XP_006347521.1	*Solanum tuberosum*
	**StDMR6-2**	XP_006347314.1	*Solanum tuberosum*
	**NtFNSI**	NP_001239794.1	*Glycine max*
	**CaFNSI**	XP_027109944.1	*Coffea Arabica*
	**PpFNSI**	XP_007218269.1	*Prunus persica*
	**PtrFNSI**	XP_002321354.3	*Populus trichocarpa*
	**TcFNSI**	EOY23715.	*Theobroma cacao*
	**CsFNSI**	XP_028118921.1	*Camellia sinensis*
	**AtDMR6**	AT5G24530.1	*Arabidopsis thaliana*
	**OsFNSI-1**	LOC_Os03g03034.1	*Oryza sativ*
	**OsFNSI-2**	LOC_Os04g49194.1	*Oryza sativa*
	**TaS5H**	ABB20895.1	*Triticum aestivum*
	**SbDMR6-1**	Sobic.001G526900.1	*Sorghum bicolor*
	**SbDMR6-2**	Sobic.001G314300.1	*Sorghum bicolor*
	**SbDMR6-3**	Sobic.006G190000.1	*Sorghum bicolor*
	**ZmFNSI-1**	Zm00001d029744	*Zea may*
	**ZmFNSⅠ-2**	Zm00001d027423	*Zea mays*
	**HvS5H**	BAJ94485.1	*Hordeum vulgare*
S3H	**SiS3H**	7G210600.1	*Setaria italic*
	**ZmS3H**	Zm00001d002564	*Zea mays*
	**OsDLO**	LOC_Os04g49210.1	*Oryza sativa*
	**HvS3H**	BAJ94485.1	*Hordeum vulgare*
	**PpS3H**	XP_007218269.1	*Prunus persica*
	**PtrS3H**	XP_002317046.3	*Populus trichocarpa*
	**AtS3H**	AT4G10500.1	*Arabidopsis thaliana*
	**AtDLO2**	AT4G10490.1	*Arabidopsis thaliana*
	**VvS3H**	XP_002267625.1	*Vitis vinifera*
	**TcDLO2**	XP_007039319.1	*Theobroma cacao*
FNSI	**SmFNSI/F3H**	XP_002985262	*Selaginella moellendorffii*
	**PaFNSI/F2H**	KJ439220	*Plagiochasma appendiculatum*
	**MeFNSI**	QEP99659.1	*Marchantia emarginata*
	**MpFNSI**	QEP99658.1	*Marchantia paleacea*
	**CjFNSI/F2H**	QEP99661	*Conocephalum japonicum*
	**CjFNSI**	QEP99662	*Conocephalum japonicum*

	**PaFNSI**	QEP99657.1	*Plagiochasma appendiculatum*
	**CcFNSI/F2H**	QEP99660.1	*Conocephalum conicum*
	**DcFNSI**	AF184270	*Daucus carota*
	**PcFNSI**	AY817680	*Petroselinum crispum*
	**AcFNSI**	DQ683350	*Aethusa cynapium*
	**PpFNSI/F3H**	XP_001780809	*Physcomitrella patens*
	**PrF3H/FNSI**	AGY80772	*Pinus radiate*
	**PsF3H/FNSI**	ABK25766	*Picea sitchensis*
	**GbF3H/FNSI**	AAU93347	*Ginkgo biloba*
	**AgFNSI**	AY817676	*Apium graveolens*
F3H	**HvF3H**	ACH42080.1	Hordeum vulgare
	**AmF3H**	AY817678	*Ammi majus*
	**PaF3H**	AY817674	*Pimpinella anisum*
	**PcF3H**	AY230248	*Petroselinum crispum*
	**AgF3H**	AY817679	*Anethum graveolens*
	**AcF3H**	DQ683351	*Aethusa cynapium*
	**MtF3H**	8g024120.1	*Medicago truncatula*
	**VvF3H**	NP001268034.1	*Vitis vinifera*
	**CsF3Ha**	KY615688	*Camellia sinensis*
	**CsF3Hb**	KY615689	*Camellia sinensis*
	**AtF3H**	NP_190692	*Arabidopsis thaliana*
	**SbF3H**	ADB66755.1	*Sorghum bicolor*
	**ZmF3H**	NM_001136803	*Zea mays*
	**OsF3H**	XM_015779149	*Oryza sativa*
	**TaF3H**	ABR13013.1	*Triticum aestivum*
	**DuF3H**	comp23389	*Desmodium uncinatum*
ANS	**NtANS**	XP_009604108.1	*Nicotiana tomentosiformis*
	**AtANS**	NP_001031700	*Arabidopsis thaliana*
	**CsANSa**	KY615704	*Camellia sinensis*
	**CsANSb**	KY615703	*Camellia sinensis*
	**VvANS**	RVW17740.1	*Vitis vinifera*
	**GmANS**	NP_001239794.1	*Glycine max*
	**MtANS**	XP_003611189.1	*Medicago truncatula*
	**DuANS**	comp23380	*Desmodium uncinatum*
	**GmLDOX**	NP_001241248.1	*Glycine max*
	**CsFLSa:**	KY615705	*Camellia sinensis*
	**CsFLSb**	KY615707	*Camellia sinensis*
	**CsFLSc**	KY615706	*Camellia sinensis*
	**GsFLS**	KHN35581	*Glycine soja*
	**FaFLS**	ABH07784	*Fragaria x ananassa*

**FLS**	**PpFLS**	AJO70134	*Prunus persica*
	**DuFLS**	comp24814	*Desmodium uncinatum*
	**AtFLS1**	AED91333	*Arabidopsis thaliana*
	**VvFLS1**	AB086055	*Vitis vinifera*
	**DuLDOX**	comp23594	*Desmodium uncinatum*
	**VvLDOX**	RVW17740.1	*Vitis vinifera*
	**MtLDOX**	XP_003601080.1	*Medicago truncatula*
